# Practical Notes and Formulæ

**Published:** 1881-08-20

**Authors:** 


### PRACTICAL NOTES AND FORMULÆ.

**Sassafras Oil as a Remedy for Rhus-Poisoning.**—Dr. H. Neeson, of Bellevue, La., writes to New Remedies the following:

Allow me to suggest the free use of oil of sassafras in these cases. I have used it for years, and have never been disappointed in a single instance. The following is my prescription:

| | |
|---|---|
| Sweet oil (fresh cream is better) | ℥j; 30.00 Gm., |
| Oil sassafras | gtt. xv; 1.00 " |

M. S. Anoint the face, hands, and all affected parts well three or four times a day.

If the bowels are constipated or there are febrile symptoms, give sulphate of magnesia as a laxative. Try it, and you will be satisfied with the results even in the most aggravated case.—*Louisville Med. News.*

**How to Cover the Odor of Iodoform.**—One of our leading pharmacists sends us the following formula, which he claims will effectually mask the odor of iodoform:

| | | | |
|---|---|---|---|
| Iodoform | ʒss; | 2.00 | Gm., |
| Oil lavender | gtt. x; | 0.30 | " |
| Alcohol | fl. ʒij; | 8.00 | fl Gm, |
| Glycerin | fl. ℥vi; | 24.00 | " M. |

Any one who recalls the many bad smelling substances which have been proposed for this purpose, will take comfort in the above.—*Ibid.*

**Treatment of Hay-Fever.**—Dr. Hermann Hager, who has observed a case of catarrh with subsequent asthmatic trouble and loss of appetite, which closely resembled the hay-fever of England and the United States, employed the following:

| | | | |
|---|---|---|---|
| ℞ | Quinidiæ | ʒijss; | 10.00 Gm., |
| | Tragacanthæ | ʒj | 4.00 " |
| | Althæa rad | gr. xv; | 1.00 " |
| | Gentian rad | ʒij; | 8.00 " |
| | Glycerin<br>Acid. hydrochloric | aa *m* cviij; | 7.00 " |

M. Make two hundred pills. Take three every two hours

The condition of the patient improved during the course of the first day, and upon the second day the patient was well. Six months afterward the attack again occurred, but yielded readily to the same treatment.

Hager thinks that hay-fever is caused more probably by the dust or spores of fungi than by the pollen of phenogamous plants.—*Pharm. Centralhalle, New Remedies.*

**Dysentery and Diarrhœa.**—We give below a number of formulæ which experience has proven to be useful in dysentery and diarrhœa. Some of them may be found in the back volumes of our Journal, but are here reproduced for the benefit of our new subscribers:

**For Acute Dysentery**—

℞ Epsom salts.................................... 1 drachm.
Water............................................ 8 ounces.
Morphine........................................ ½ grain.

M. S. Take one tablespoonful every hour until the character of the discharge changes, and then once every two or three hours to keep up the impression. After pursuing this treatment one or two days, the patient keeping quiet and using a little boiled milk as a diet, the case recovers or may be arrested by administering an opiate.

**Another Excellent Remedy for Dysentery**, especially of the billious form, is the following—

℞ Corrosive sublimate........................ 1 gr.
Water,.............................................. 1 pint.

Dose for an adult from half to one teaspoonful every three hours until the action of the liver is restored, when with or without an opiate the case usually recovers, usually within 48 hours. Salivation is not likely to occur in the use of this remedy unless long continued.

**Chloral in Dysentery.**—When great tormina exists the following seldom fails to give relief in dysentery:

℞ Chloral.............................................. 10 grs.
Thin starch......................................... 2 ounces.

Use by enema. The pain is relieved usually for several hours, the patient sleeping or resting quietly, when if necessary, it may be repeated. If this remedy be first preceded by castor oil or epsom salts to remove all the offending matter, it will not unfrequently arrest the disease at once.

**Subnitrate of Bismuth** in twenty grain doses for an adult is a good remedy in dysentery; or for children in cases of dysentery or dysenteric diarrhœa while teething, the following may be used:

℞ Subnitrate bismuth,
Pepsin,
Tannin,
Pulv. cinnamon, aa.......................... 2 grs.

As one dose every three hours in a child over one year old.

**For Dysenteric Diarrhœa** in children, especially when teething:

℞ Subnitrate bismuth ........................ 2 drachms,
Syr. ginger...................................... 1 ounce,
Mucilage of gum arabic.................... 3 drachms,
Creosote.......................................... 5 to 10 drops,
Camph. water to make..................... 6 ounces.

Dose, one teaspoonful every three hours for a child one to two years old.

**Sulphate of Zinc as a Caustic.**—Prof. Simpson, in Annals of Surgery, sums up its advantages, as compared with other caustics, as follows :

1. Its powerful escharotic action.
2. The rapidity of its action.
3. Its great simplicity and manageableness.
4. Its facility of application.
5. Its non-tendency to deliquesce or spread.
6. Its perfect safety.
7. Its efficacy.

He speaks hesitatingly as to the seventh statement, but adds that he has seen not only the surface of cancroid and cancerous ulcers speedily and perfectly excavated by its application, but the surrounding characteristic induration became at the same time rapidly absorbed, and the remaining wound speedily cicatrices. The slough is of a white color and comes off on the fifth or sixth day. A mass or paste is made by mixing

Sulph. Zinc.........................................
Sulphuric acid.................................... aa q. s.

**Headache of Dyspepsia.**—If the headache is accompanied with atonic dyspepsia, and there is a clean tongue with weight and oppression of the epigastrium, nitro-muriatic acid will be found serviceable before meals or three times per day. Dr. Day recommends the following formula in his work On Headache:

| | | | |
|---|---|---|---|
| ℞ Tinct. nuc. vom..................... | aa ʒj; | 4.00 fl. Gm. |
| Acid nitr. dil........................ | | |
| Acid hydrochl dil........................ | ʒij; | 8.00 | " |
| Tinct. aurant............................ | ʒvj; | 24.00 | " |
| Aqæ puræ ad............................ | ℥vj; | 180.00 | " |

Misce. A tablespoonful in a wineglassful of water three times a day. —*Medical Press and Circular.*

**Digitalis.**—The poisonous effects of this remedy as given in Taylor's Jurisprudence is as follows : A young man swallowed a strong decoction of fox-glove by mistake for a purgative ; he was soon seized with vomiting, abdominal pains and purging. He died twenty-two hours after taking the poison. The pulse was slow and irregular. A common effect of the poison is to produce great depression of the heart's action. What will this remedy do in an enfeebled condition of the heart? We all know that when the tincture is given in doses of one-half to one drop every two hours, that it is one of our best heart tonics.—*Ex.*

**Stramonium.**—I have as yet had but one indication for this remedy, and that is very direct and plain, said Dr. Gerard before the Massachusetts Eclectic Medical Society. In irritation of the neck of the bladder and the urethra in woman, with burning pains and a frequent desire to urinate, I have found stramonium to give relief usually in a few hours, or two or three days at the longest. Dose, one to three drops of the tincture every two or three hours.

**Improved Chalked Mixture for Infantile Diarrhœa.**—

| | | |
|---|---|---|
| ℞ | Prepared chalk........................................... ... | |
| | Loaf sugar, pulv.............................................aa | ½ ounce. |
| | Glycerine......................................................... | 2 drachms. |
| | Cinnamon water.............................................. | 2½ ounces. |
| | Creosote.......................................................... | 3 drops. |

Dose, a teaspoonful every three hours. This preparation is more effectual than the old formula, and will not sour by keeping.

**Cholera Infantum.**—

| | | |
|---|---|---|
| ℞ | Creosote.................... .......................... .................... | gtt. j. |
| | Lime water........................................................................ | ℥ ij. |

One teaspoonful with a teaspoonful of milk, breast milk if a suckling infant, repeated every one to two hours. Useful when vomiting is troublesome.—*Naphey.*

**Gelseminum.**—Gelseminum is recommended in irritability of the nervous system with a determination to the brain, causing flushed face, contracted pupils, supra-orbital neuralgia, and is one of our best remedies. In hysterical spasms and in many cases of spermatorrhœa, it is very efficient.—*Chicago Med. Times.*

**Bichromate of Potash.**—This remedy has a peculiar effect upon mucous membranes. It so changes the functions of the mucous follicles as to cause them to secrete a tough, ropy mucus from the nose, mouth and throat. In mucous and membranous croup, better results are obtained from its use than from any other remedy. In chronic Laryngitis and bronchitis, with tough, stringy expectoration, it is one of our most useful remedies. Use the first decimal trituration, adding two or three grains to half a tumblerful of cold water, or enough to make the water a little yellow. Dose, a teaspoonful every half hour or hour.—*Ex.*

**Chlorate Potassa.**—If there is a specific in medicine this remedy is certainly one. In all cases of a fœtid breath caused by a vitiated condition of the stomach and bowels, I never have known it to fail in a single case; of course it will not relieve in cases caused by decayed teeth.—F. L. Gerald, M.D., *Med. Times.*

**Belladonna in Dysentery.**—Two to four drops of belladonna (tincture or fluid extract) be given every two to four hours until the griping pains and tenesmus ceases, and then gradually diminished in dose, will often relieve the most obstinate cases of acute dysentery.

**Primary Cancer of the Pancreas.**—Dr. Kennig reports, in the Petersburg Medical Woch., February 2, a minute history of a case of this rare affection occurring in a woman aged fifty-three.—*Med. News and Abstract.*

